# Selective nerve root injection of ozone for the treatment of phantom limb pain: Three case reports

**DOI:** 10.1097/MD.0000000000019819

**Published:** 2020-04-17

**Authors:** Juanhong Li, Tianzuo Li, Guiying Li, Hongfu Liu, Xiaogai Zhang

**Affiliations:** a Department of Pain Medicine, Beijing Shijitan Hospital, Capital Medical University; b Department of Nuclear Medicine, Beijing Geriatric Hospital, Beijing, China.

**Keywords:** amputation, case report, ozone injection, phantom limb pain

## Abstract

**Rationale:**

Phantom limb pain (PLP) refers to a common complication following amputation, which is characterized by intractable pain in the absent limb, phantom limb sensation, and stump pain. The definitive pathogenesis of PLP has not been fully understood, and the treatment of PLP is still a great challenge. Till now, ozone injection has never been reported for the treatment of PLP.

**Patient concerns:**

We report 3 cases: a 68-year-old man, a 48-year-old woman, and a 46-year-old man. All of them had an amputation history and presented with stump pain, phantom limb sensation, and sharp pain in the phantom limb. Oral analgesics and local blocking in stump provided no benefits.

**Diagnosis:**

They were diagnosed with PLP.

**Interventions:**

We performed selective nerve root ozone injection combined with ozone injection in the stump tenderness points.

**Outcomes:**

There were no adverse effects. Postoperative, PLP, and stump pain were significantly improved. During the follow-up period, the pain was well controlled.

**Lessons:**

Selective nerve root injection of ozone is safe and the outcomes were favorable. Ozone injection may be a new promising approach for treating PLP.

## 1 Introduction

Phantom limb pain (PLP) refers to a common complication following amputation, which is characterized by intractable pain in the absent limb, phantom limb sensation, and stump pain. It affects approximately 60% to 80% of all post-amputation patients.^[1]^ As the pain is usually refractory, PLP severely hinders the patients’ functional and psychological rehabilitation and considerably impairs individual quality life.

The definitive pathogenesis of PLP has not been fully understood, and the treatment of PLP is still a great challenge.^[2]^ The current therapeutic strategies for managing PLP include 3 categories: pharmacological interventions, such as tricyclic antidepressants, anticonvulsants, calcitonin, opioids, and serotonin–norepinephrine reuptake inhibitors; non-pharmacological and non-invasive therapies, such as repetitive transcranial magnetic stimulation, visual feedback, behavior reflex, and hypnotherapy; and invasive surgeries, such as patch repair for the amputation stump, selective rhizotomy, spinal radiculectomy, and electrical stimulation therapy.^[1,3,4]^ The efficacy of these approaches remains indeterminate.

Selective nerve root injection of ozone has been used clinically for more than half a century. Recently, Bonetti et al^[5]^ and Gallucci et al^[6]^ attempted to inject ozone, steroid, local anesthetics, or their mixture via an intradiscal or intraforaminal approach for treating lower back pain and sciatica; they proposed ozone injection is an effective and safe modality to relieve the pain. However, till now, ozone injection has never been reported for the treatment of PLP.

Herein, we described 3 cases with PLP in which the pain was well controlled by selective nerve root injection of ozone. Informed consent has been obtained from the patients for the publication of this case report.

## 2 Case report

### 2.1 Case 1

A 68-year-old man presented to us with a 38-year history of PLP. Forty years ago, he underwent left proximal-femur amputation due to an accident at work, and 2 years later he developed stump pain, phantom limb sensation, and stinging pain in the phantom left lower limb (mainly localized in the ankle, toe joints, and toes). The pain was severe and attacked several times daily, each episode lasting from several minutes to hours. Oral ibuprofen, carbamazepine, and paracetamol had been prescribed but provided no benefits. In other institutions, the patient was treated with steroid blocking in the stump several times, while the treatment remained ineffective. Ten years ago, in the Department of Orthopedics, the stump neuroma was surgically resected and a bone plasty was performed in the stump. Postoperatively, the pain was relieved; nevertheless, 5 months later, the pain reoccurred. In the last 1 month prior to admission, the pain was exacerbated and radiative to the left ankle. Physical examination showed 2 tenderness points in the stump scar and the Tinel sign was positive. The Visual Analogue Scale (VAS) score for the stump pain and the pain in the phantom left lower limb was 8 and 9, respectively.

The patient was diagnosed as PLP, and a 3-stage ozone injection was scheduled. For the first-stage treatment, a selective nerve root injection of ozone with ozone injection in the stump tenderness points was performed under local anesthesia. In the right lateral decubitus, the needle was punctured into the left lateral recess at the L4 level, and neurophysiological monitoring confirmed the localization. A 5 mL mixture (including saline for 3.75 mL, 2% lidocaine hydrochloride for 1.25 mL, triamcinolone acetonide for 5 mg, and cobamamide for 0.75 mg) was injected followed by ozone (30 μg/mL) for 20 mL. Computed tomography showed the ozone was well diffused in the target area. In the supine position, the stump tenderness points were injected with a 5 mL mixture (as mentioned above) and ozone (30 μg/mL) for 10 mL. The VAS score for the stump pain and the pain in the phantom left lower limb was 5 and 0, respectively. In the following month, the patient underwent the second-stage ozone injection using the same method as the previous treatment. The VAS score for the stump pain and the pain in the phantom left lower limb was 4 and 0, respectively. In the third month, we injected the ozone into the stump tenderness points and the nerve-entrapment point (the point with positive Tinel sign; 8 cm cephalad to the stump tenderness point on the ischiadic nerve). After the 3-stage ozone injection, the VAS score for the stump pain and the pain in the phantom left lower limb was 2 and 0, respectively. There were no adverse effects. After a follow-up of 6 months, the pain remained unchanged.

### 2.2 Case 2

A 48-year-old woman presented with a 12-year history of PLP. Thirteen years before, she underwent right lower extremity amputation due to a traffic accident. One year later, she developed stump pain, phantom limb sensation, and stinging pain in the phantom right lower limb (mainly localized in the toe joints and halluces). The pain was sharp like cutting or discharging, which attacked several times per day, and each episode lasted from several minutes to hours. Oral ibuprofen and paracetamol, as well as surgical blocking in the stump, had been attempted but no efficacy was noted. Physical examination showed 3 tenderness points in the stump scar and the Tinel sign was positive. The VAS score for the stump pain and PLP was 8 and 9, respectively. A 3-stage ozone injection was performed according to the scheme as described in Case 1. The postoperative course was uneventful. Six months later, the follow-up VAS score for the stump pain and PLP was 3 and 1, respectively.

### 2.3 Case 3

A 46-year-old man presented with a 10-year history of PLP. Eleven years before, she underwent left lower extremity amputation due to a traffic accident. One year postoperatively, she developed stump pain, phantom limb sensation, and stinging pain in the phantom left lower limb (mainly localized in the ankle). The pain was sharp like discharging, which attacked 3 to 5 times per day, and each episode lasted from several minutes to hours. Oral ibuprofen and paracetamol, as well as surgical blocking in the stump, had been attempted, while there was no efficacy. Physical examination showed 2 tenderness points in the stump scar and the Tinel sign was positive. The VAS score for the stump pain and PLP was 7 and 9, respectively. A 3-stage ozone injection was performed according to the scheme as described in Case 1. The postoperative course was uneventful. After a follow-up period of 6 months, the VAS score for the stump pain and PLP was 3 and 0, respectively.

The detailed clinical profiles were summarized in Table 1.

**Table 1:**
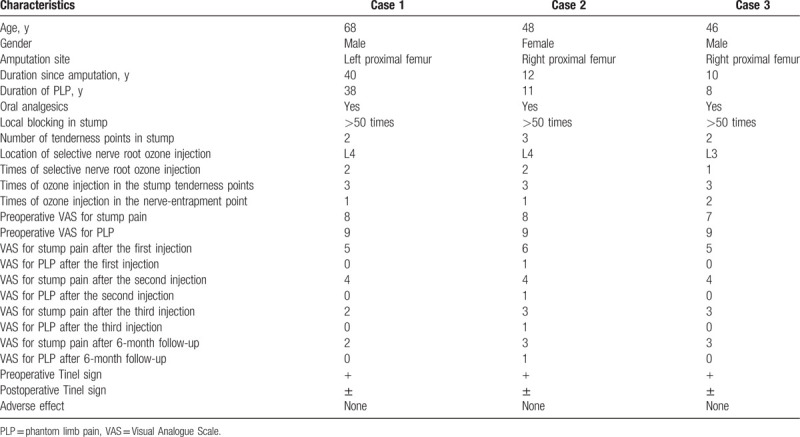
Summary of clinical profiles of 3 patients with PLP.

## 3 Discussion

The pathogenesis of PLP has not been completely clarified. Previous evidence showed that reactive oxygen species (ROS; such as hydroxyl radicals) and reactive nitrogen species (RNS; such as peroxynitrite) are closely associated with inflammatory pain, neuropathic pain, visceralgia, and chemotherapy-induced pain.^[7,8]^ Especially, disturbance of the activated oxygen metabolism is a major contributor to chronic neuropathic pain.^[9]^ The damage in peripheral nerves produced excess ROS which exceeds the body's scavenging capacity, resulting in mitochondrial dysfunction and further increasing the ROS release. ROS can reduce the presynaptic inhibitory transmission of the spinal cord and activate the N-methyl-D-aspartic acid (NMDA) and α-amino-3-hydroxy-5-methyl-4-isoxazole-propionic acid (AMPA) receptors, which alter synaptic plasticity and induce central sensitization, leading to neuropathic pain. Additionally, the overproduction of mitochondrial ROS induced by peripheral nerve injury or inflammation disrupts the function of superoxide dismutase analogues which can suppress the pain.^[10]^ In short, metabolic disturbance of ROS plays an important role in the development and progression of chronic neuropathic pain, and inhibition of the ROS level may be a promising approach for reliving PLP.^[11]^

Ozone, also known as reactive oxygen, is composed of 3 oxygen atoms, and it is an unstable gas with a distinctively pungent smell. Ozone has an oxidation capacity, and it is safe for clinical applications. Numerous data have shown that ozone can eliminate oxygen free radicals by activating antioxidant enzymes, and it can maintain and restore the oxidation–antioxidant balance. Ozone interacts with biological molecules via ozone-initiated reactions and lipid oxidation reactions. Recently, ozone has been widely used for clinical analgesia, which is related to its antioxidation effects.^[12]^ In our previous study, we found injection in the left lateral recess at the L4 level would lead to the optimal ozone diffusion.^[13]^ The dosage and frequency of ozone injection were determined according to our clinical experience and previous evidence.^[14]^ No other pain or steroid medications were administrated during the ozone treatment in these 3 cases. The contraindications of ozone therapy include: allergy to ozone, hyperthyroidism, deficiency of Glucose-6-phosphate Dehydrogenase (G6-PD), spinal canal stenosis, lateral recess stenosis, vertebral slippage, hemorrhagic disease, and severe hepatorenal insufficiency.

PLP is a special subtype of chronic neuropathic pain, and it is usually companied with phantom limb sensation and stump pain. The anatomical basis of PLP involves multifarious structural abnormalities in the sensory afferent system including peripheric receptors, sensory afferent fibers, and spinal conduction pathways. Moreover, PLP is reported to be closely related to psychological factors.^[2]^ The physiopathologic correlation between PLP and stump pain remains unclear; nevertheless, previous studies indicated remission of stump pain can simultaneously relieve the PLP, and abnormal input of primary afferent nerves plays a crucial role in the pathogenesis of PLP.^[15]^ Thus, we speculate that relieving stump pain may be one of the core steps for the treatment of PLP. In this study, all 3 patients had attempted surgical therapies such as steroid blocking in the stump, resection of stump neuroma and stump plasty, but no benefits were gained. Both the PLP and stump pain were well controlled by the selective nerve root injection of ozone combined with ozone injection in the stump tenderness points. Previous clinical studies have indicated that dorsal root ganglion may be the target for treating PLP, as dorsal root ganglion manifests as abnormal autonomic activities and increased sensibility to mechanical and neurochemical stimuli.^[16]^ In our cases, ozone injection exhibited satisfying efficacies, which also supports the above hypothesis. Ozone injection may be a new promising approach for treating PLP.

## Author contributions

**Conceptualization:** Juanhong Li, Xiaogai Zhang.

**Data curation:** Juanhong Li, Tianzuo Li, Guiying Li, Hongfu Liu, Xiaogai Zhang.

**Investigation:** Guiying Li.

**Methodology:** Juanhong Li, Tianzuo Li, Guiying Li.

**Supervision:** Juanhong Li, Xiaogai Zhang.

**Writing – original draft:** Juanhong Li.

**Writing – review & editing:** Juanhong Li, Hongfu Liu.
